# A closer look to apathy

**DOI:** 10.1192/j.eurpsy.2021.1266

**Published:** 2021-08-13

**Authors:** D. Silva, R. Martins, F. Polido, M.D.C. Cruz

**Affiliations:** Psychiatry, Centro Hospitalar Universitário do Algarve, Portimão, Portugal

**Keywords:** apathy

## Abstract

**Introduction:**

Apathy is a neuropsychiatry syndrome, conceptualised as a loss of motivation free of altered consciousness, cognitive impairment or emotional distress, associated with a wide range of brain disorders such as Dementia, Major depression and schizophrenia. Even though under-recognized and under-diagnosed, apathy hardly appears uncommon. Its reported frequency in various neurologic and psychiatric conditions varies widely, from less than 10 to over 80%, reflecting differences in population characteristics and assessment procedures.

**Objectives:**

The aim of this article is to review the concept of Apathy and clarify its nosology, pathopshysiology and treatment.

**Methods:**

An online bibliographic search was carried out on PubMed and Medline using “Apathy” as a term.

**Results:**

The literature reviewed shows that apathy is a multi-dimensional clinical construct with a current definition and validated diagnostic criteria. Analysis reveals that apathy is strongly associated with disruption particularly of anterior cingulate cortex (ACC), ventral striatum (VS) and nucleus accumbens (N acc). Remarkably, these changes are consistent across clinical disorders and imaging modalities, playing a crucial role in normal motivated behaviour.

**Conclusions:**

The neuromodulator dopamine is heavily implicated in ACC and VS. Therapeutically, numerous small studies suggest that psychostimulants, dopaminergics, and cholinesterase inhibitors may benefit those manifesting this syndrome. However, no adequately powered, randomized controlled trials have reported success and no medication have ever been approved for this disorder Further research is needed to help understand the functional neuroanatomy, neuromodulators involved and possible treatment options of this clinical construct.

